# Development, Optimization, and In Vitro/In Vivo Evaluation of Azelaic Acid Transethosomal Gel for Antidermatophyte Activity

**DOI:** 10.3390/antibiotics12040707

**Published:** 2023-04-05

**Authors:** Ali M. Nasr, Noha M. Badawi, Yasmine H. Tartor, Nader M. Sobhy, Shady A. Swidan

**Affiliations:** 1 Department of Pharmaceutics, Faculty of Pharmacy, Port Said University, Port Said 42526, Egypt; 2 Department of Pharmaceutics and Industrial Pharmacy, Faculty of Pharmacy, Galala University, New Galala 43713, Egypt; 3 Department of Pharmaceutics and Pharmaceutical Technology, Faculty of Pharmacy, The British University in Egypt, El-Sherouk City, Cairo 11837, Egypt; 4 The Centre for Drug Research and Development (CDRD), Faculty of Pharmacy, The British University in Egypt, El-Sherouk City, Cairo 11837, Egypt; 5 Department of Microbiology, Faculty of Veterinary Medicine, Zagazig University, Zagazig 44511, Egypt; 6 Department of Animal Medicine, Faculty of Veterinary Medicine, Zagazig University, Zagazig 44511, Egypt

**Keywords:** transethosomes, azelaic acid, antidermatophyte, thin film hydration, guinea pig model, *Trichophyton mentagrophytes*, *Microsporum canis*

## Abstract

Treatment of dermatophytosis is quite challenging. This work aims to investigate the antidermatophyte action of Azelaic acid (AzA) and evaluate its efficacy upon entrapment into transethosomes (TEs) and incorporation into a gel to enhance its application. Optimization of formulation variables of TEs was carried out after preparation using the thin film hydration technique. The antidermatophyte activity of AzA-TEs was first evaluated in vitro. In addition, two guinea pig infection models with *Trichophyton* (*T.*) *mentagrophytes* and *Microsporum* (*M.*) *canis* were established for the in vivo assessment. The optimized formula showed a mean particle size of 219.8 ± 4.7 nm and a zeta potential of −36.5 ± 0.73 mV, while the entrapment efficiency value was 81.9 ± 1.4%. Moreover, the ex vivo permeation study showed enhanced skin penetration for the AzA-TEs (3056 µg/cm^2^) compared to the free AzA (590 µg/cm^2^) after 48 h. AzA-TEs induced a greater inhibition in vitro on the tested dermatophyte species than free AzA (MIC_90_ was 0.01% vs. 0.32% for *T. rubrum* and 0.032% for *T*. *mentagrophytes* and *M. canis* vs. 0.56%). The mycological cure rate was improved in all treated groups, specially for our optimized AzA-TEs formula in the *T. mentagrophytes* model, in which it reached 83% in this treated group, while it was 66.76% in the itraconazole and free AzA treated groups. Significant (*p* < 0.05) lower scores of erythema, scales, and alopecia were observed in the treated groups in comparison with the untreated control and plain groups. In essence, the TEs could be a promising carrier for AzA delivery into deeper skin layers with enhanced antidermatophyte activity.

## 1. Introduction

Studies have shown that the greatest number of dermatological disorders occur as superficial fungal infections at a high rate in developing countries, and can in some cases lead to death [1]. Dermatophytic infection affects millions of people worldwide every year [2]. Recent years have seen a rise in the incidence of fungal topical infections which have proven to face significant obstacles in their treatment. Dermatophytosis is among the most prevalent forms of infectious diseases globally, accounting for almost 25% of all skin mycoses worldwide [3,4]. Dermatophytosis is a superficial fungal infection of the keratinized structures including skin, hair, nails, or animal hoof and claws caused by a dermatophyte mold which is most commonly of the Trichophyton and Microsporum genus. The infection can be transmitted to humans by anthropophilic species, geophilic species from the soil, or zoophilic species through contact with animals [5].

Treatment of dermatophytosis might be quite challenging and needs a cautious approach in certain population groups including pregnant women, children, and the elderly. Recently, there is a tendency to limit treatment to topical therapies owing to their negligible systemic absorption. Future research on newer medications or topical drug formulations with a “depot” impact may contribute to a reduction in the treatment duration as well as recurrences [4]. The main difficulties with the treatment of dermatological fungal infections are how to bypass the stratum corneum (SC) barrier, the long time required for treatment, and recurring infection. Due to the presence of tight junctions and the highly organized structure of the SC, dermatophytosis treatment is complicated. Efficient treatment requires the presence of antifungal drugs with a high concentration on the epidermis and dermis [6]. There is a preference for the use of topical antifungal drugs over systemic ones since the drug is delivered directly to the affected site leading to fewer adverse effects [7].

Further approaches were carried out to develop a drug delivery system that enables various antifungal drugs to bypass the SC’s barrier function. Among these novel drug delivery systems, lipid-based nanoformulations such as liposomes have proven to be promising drug carriers for dermatological delivery and attracted the attention of many researchers in this field [8]. It should be noted, however, that the liposomal rigid structure and their large size have limited drug diffusion into deeper skin layers [9]. As a consequence, an improvement over liposomal strategy was developed recently and novel approaches of lipid-based vesicles such as the ultra-deformable liposomes (transfersomes), ethosomes, and transethosomes (TEs) have been designed as enhanced versions of liposomes [10]. Transfersomes are liposomal formulations that contain edge activators. An advantage of this approach is that these edge activators destabilize the liposomal lipid bilayer and improve the flexibility of liposomes [11]. Ethosomes, on the other hand, are novel flexible malleable vesicles that consist of phospholipids, water, and a high concentration of ethanol [12]. Ethosomes enhance the penetration rate of drugs across the SC and can bypass this critical barrier where ethanol increases the vesicles’ flexibility by fluidizing the lipid bilayers, allowing them to squeeze through the skin pores which are much smaller than their original diameters [13]. Thus, if we can formulate a system that combines the benefits of transfersomes and ethosomes, it will be an invaluable carrier in delivering the drugs to deeper skin layers. This was achieved recently with the development of nanovesicles TEs. TEs are vesicular systems containing phospholipid (e.g., soy lecithin), water, and high concentration of ethanol along with an edge activator (EA) or permeation enhancer [14]. The EA plays an important role as it preserves the integrity of the vesicles when going across small channels, thus increasing stability and the deformity is consequently procured [15]. The particle size of TEs is the most important factor for their skin penetration. Recent studies stated that a size below 500 nm is necessary for the efficient diffusion of nanocolloids through the SC [16]. Additionally, hydrogels have been widely utilized as potential strategies for drug delivery. This could be attributed to their outstanding biocompatibility, biodegradability, wide mechanical properties that match various soft tissues in the human body, and controllable drug release capabilities [17,18,19].

Azelaic acid (AzA) is straight-chained dicarboxylic acid approved for acne vulgaris and inflammatory (papulopustular) rosacea treatment. Owing to its poor water solubility, about 0.24 g/100 mL at 25 °C, as well as limited skin penetration across the SC, various formulations have been developed, including liposome, microemulsion, ethosomes, and liquid crystal [20]. Since relatively little is known about the antidermatophyte activity of AzA, we investigated for the first time its in vitro and in vivo effects on dermatophyte species in comparison with its nano transethosomal formulation (AzA-TEs) and itraconazole as a positive control.

To date, no attempt has been made to investigate the potential of TEs as carriers for AzA, and no trials have been carried out to examine the antidermatophyte effect of the AzA-TEs nanocarriers. The current investigation aimed to develop a nanotransethosomal gel of AzA for efficient cutaneous delivery, in order to improve its biopharmaceutical profile and enhance therapeutic efficacy as an antifungal for dermatophytosis treatment. The overall study was carried out in two steps. AzA-TEs were developed and formulated in the first step using the quality by design methodology. Additionally, the morphology, vesicle size, zeta potential, polydispersity index, and physical state of the optimized formulation were characterized. The second stage included the incorporation of the optimized TEs vesicles into a Carbopol-based gel for enhanced skin adherence. Two guinea pig models are performed in the in vivo study of AzA-TEs. Various dermatophyte species including *T. mentagrophytes* and *M. canis* have been utilized successfully in the current study animal models as they cause acute inflammatory infections which are easy to be clinically evaluated.

## 2. Materials and Methods

### 2.1. Materials

Azelaic acid was a kind gift from Medical Union Pharmaceuticals (MUP, Ismailia, Egypt), Labrafil M 1944 SC, and was kindly gifted by Gattefossé, (Saint-Priest, France). Soy leci-thin, Sodium deoxycholate (SDC) and Dialysis tubing cellulose membrane (molecular weight cut off 12,000–14,000 Dalton) were purchased from Sigma Aldrich Chemical Co. (St. Louis, MO, USA). Oleic acid and Carbopol 934 were purchased from El-Nasr Pharmaceutical Company (Cairo, Egypt). Dichloromethane and ethanol were purchased from Fisher Scientific (Loughborough, UK). All other materials were of analytical grade.

### 2.2. Methods

#### 2.2.1. Experimental Design

A 2^3^ full factorial design was generated for the optimization of AzA-TEs using the Design-Expert 11 program (Stat-Ease, Inc., Minneapolis, MN, USA). Eight runs were created in this model by the design. Three independent variables were selected to be studied including SAA type (X1), SAA:Lecithin ratio (X2), in addition to ethanol concentration% (X3). The outcome responses for this design were particle size (PS) (Y1), zeta potential (ZP) (Y2), and entrapment efficiency (EE%) (Y3). Table 1 presents the independent and dependent variables utilized in the current study as well as their levels and desirability constraints.

#### 2.2.2. Preparation of AzA-Loaded TEs

AzA-TEs vesicles were prepared via the thin-film hydration method [21]. Precisely weighed amounts of Soy Lecithin, SAA, oleic acid, and AzA were added to 10 mL dichloromethane in a long-necked round-bottom flask as shown in Table 2. The solvent mixture was then evaporated under vacuum at 60 °C and 90 rpm speed via Heidolph rotary evaporator (P/N Hei-AP Precision ML/G3, Schwabach, Germany). The vacuum was applied until the solvent was evaporated totally and a thin film was formed on the round flask wall. The dried film was then hydrated by adding 10 mL distilled water containing ethanol, at 60 °C which is above the lipid phase transition temperature (Tc), and stirred for 45 min. Finally, the prepared dispersion was sonicated for 10 min using a probe sonicator (Vibra Cell-Sonics Material, 130 W, 20 kHz, Newtown, CT, USA) at 70% amplitude to obtain the AzA-loaded TEs. The dispersed vesicles were then stored for further investigations at 4 °C.

#### 2.2.3. Characterization and Optimization of the Prepared AzA-Loaded TEs Formulations

##### Particle Size (PS), Polydispersity Index (PDI), and Zeta Potential (ZP) Measurement

The average PS, PDI, and ZP of the fabricated AzA-TEs formulations were attained via dynamic light scattering technique using a Zeta-sizer 3000 PCS (Malvern Instruments Ltd., Worcestershire, UK). Before analysis, the samples were diluted with de-ionized water. The obtained values were the means of triplicate measurements at 25 ± 1 °C [22].

##### Entrapment Efficiency (EE%) Measurement

EE% of AzA-TEs were calculated using the centrifugation method. Firstly, the vesicular dispersions of the prepared formulae were centrifuged at 20,000 rpm at a temperature of 4 °C for 2 h utilizing a cooling centrifuge (Sigma 3K 30, Osterede am Harz, Germany). After that, the supernatant was separated and collected to be analyzed λ_max_ 204 nm using a UV-Vis spectrophotometer (Shimadzu UV1650 Spectrophotometer, Kyoto, Japan) [23]. Determination of EE% was carried out using the following equation [24]:

$$\mathrm{EE}\%=\left(\frac{\mathrm{Total}\;\mathrm{AzA}\;\mathrm{concentration}\;-\;\mathrm{Free}\;\mathrm{AzA}\;\mathrm{concentartion}}{\mathrm{Total}\;\mathrm{Aza}\;\mathrm{concentration}}\right)\times \;100\%$$

#### 2.2.4. Optimization of Formulation Variables

For selecting the optimized formula to be subjected to further studies, the desirability function that expects the optimum levels of the response variables was calculated. The principle for choosing the optimum formula was attaining the least PS and the highest ZP and EE%.

##### Transmission Electron Microscopy

The morphology of the optimized AzA-TEs was inspected via a transmission electron microscope (Joel JEM 1230, Tokyo, Japan). Firstly, the application of the vesicular dispersion was carried out on a carbon-coated copper grid and left to be dried in order to produce a thin film. A drop of 2% aqueous solution of uranyl acetate was placed for 1 min. The remaining solution was swept and the sample was air-dried at room temperature and then viewed and photographed [25].

##### Fourier Transform Infrared (FTIR) Spectroscopy

Infrared spectra of the free drug AzA, medicated optimized formula and the plain formula (without drug) were attained using FTIR spectrophotometer VERTEX 70 (Bruker Corporation, Karlsruhe, Germany) [26]. The spectra were analyzed in the range of 500 to 4000 cm^−1^. The spectrum of air was used as a background before the analysis of each sample. Sample spectra in addition to the background were taken in a room with a temperature of 22–24 °C, at a spectral resolution of 4 cm^−1^. For each measurement, 32 scans were carried out.

##### In Vitro Release of AzA from the Optimized Formula

The in vitro release behavior of the AzA-TEs optimized formula was performed via the dialysis bag technique and compared with free AzA [27]. Before using the cellulose dialysis bag, it was soaked in phosphate-buffered saline pH 7.4 for 24 h. AzA-Tes equivalent to 10 mg of AzA was accurately weighed and the same amount of free AzA was dispersed in phosphate-buffered saline pH 7.4 and then subjected to 5 min vortexing. Afterward, samples were placed in the dialysis bags, and the bag’s ends were tightly closed. The bags which acted as the donor cell were kept in a phosphate-buffered saline pH 7.4 (50 mL) that represented the receptor cell and then placed in a shaker water bath (WSB-18, Dahan Scientific Co., Ltd., Seoul, Korea), with a rotation speed of 50 rpm and temperature of 37 ± 0.5 °C. Ethanol (30% *v*/*v*) was added to the phosphate-buffered saline pH 7.4 to maintain the sink condition [28]. Aliquots were withdrawn at different time intervals from 1 to 48 h, substituted with fresh buffer to maintain the sink condition, and then analyzed spectrophotometrically at 204 nm. Triplicate samples were analyzed and the average concentration was utilized.

##### Ex Vivo Permeation of AzA from the Optimized Formula

Mouse skin was utilized as a model to simulate human skin [29]. The ex vivo permeation experiment was performed via Franz diffusion cells with a diffusional area of 1.76 cm^2^ using the same conditions of the in vitro drug release study, except that the cellulose membrane was substituted by dorsal hairless mouse skin [27,29]. The sacrifice of male mice (weighing between 25–30 g) was first carried out, followed by hair removal, and excision of the ventral and dorsal abdominal skins. Afterward, tweezers were utilized to get rid of the subcutaneous fat, and the skin membrane fragments were washed with buffer. Subsequently, the skin fragments were kept at −20 °C until they were used [29]. The skin was placed between the two compartments (receptor and donor) of the Franz cell after being defrosted and soaked in phosphate-buffered saline pH 7.4. Following, 25 mL of phosphate-buffered saline pH 7.4 was transferred to the receptor compartment and then magnetically stirred in the water bath (37 ± 1 °C). The optimized formula was placed in the donor compartment. Then, samples (1 mL) were withdrawn from the receptor medium at different time intervals (from 1 to 48 h), and the drug concentrations were spectrophotometrically measured at λmax 204 nm. After withdrawing each sample, an equivalent volume of the fresh buffer solution was substituted. All samples were compared to a blank (without drug) to avoid any interference. This experiment was performed in triplicate and the average concentrations were used. The permeation flux (Jmax) at 48 h and the enhancement ratio (ER) were calculated according to the following equations [30]:

$$\mathrm{Jmax}=\left(\frac{\mathrm{Amount}\;\mathrm{of}\;\mathrm{drug}\;\mathrm{permeated}}{\mathrm{Time}\;\times \;\mathrm{Area}\;\mathrm{of}\;\mathrm{the}\;\mathrm{membrane}}\right)\;\mathrm{ER}=\left(\frac{\mathrm{Jmax}\;\mathrm{of}\;\mathrm{the}\;\mathrm{nanovesicles}}{\mathrm{Imax}\;\mathrm{of}\;\mathrm{drug}\;\mathrm{suspension}}\right)$$

#### 2.2.5. Formulation of AzA and AzA-TEs Gels

AzA suspension and AzA-TEs optimal formula were converted into gels to enhance the skin application. The optimized formula and the free drug suspension were added to Carbopol gel and then mixed with the assistance of a magnetic stirrer (Thennolyne Corporation, Dubuque, IA, USA) to form gels with good consistency and final AzA concentration of 0.5% [31].

#### 2.2.6. Evaluation of Antidermatophyte Activity

The antidermatophyte activity of the optimized AzA-TEs formulation was evaluated both in vitro and in vivo.

##### Dermatophyte Clinical Isolates

*T. mentagrophytes* and *T. rubrum* (n = 10 of each) that were isolated from cases of tinea corporis and *M. canis* (n = 10) recovered from tinea capitis and ringworm lesions in cats were included. Isolates were identified based on their macro- and micro-morphological, and physiological characteristics, as previously described [32]. A suspension was prepared in sterile saline from colonies of each isolate grown on mycobiotic agar medium (CONDA, Madrid, Spain) at 25 °C for a week, and adjusted to 10^6^ CFU/mL using a hemocytometer.

##### In Vitro Evaluation of Antidermatophyte Activity of AzA-TEs

The antidermatophyte activity of AzA-TEs was evaluated using an agar well diffusion test against isolates of *T. mentagrophytes*, *T. rubrum*, and *M. canis* [33]. The fungal suspensions (10^6^ CFU/mL) were evenly distributed on the surface of mycobiotic agar medium plates. With a sterile cork borer, 6 mm diameter wells were cut in the agar plate. Then, 100 µL of AzA-TEs formula (5 mg/mL), and free AzA were dissolved in 10% tween 20 (Sigma Aldrich, St. Louis, MO, USA) and adjusted to 5 mg/mL, gel (1.25 mg/mL) was placed in wells. The test was performed in triplicate and a negative control (tween 20) and a positive control (10 µg itraconazole disc; Hi-Media Laboratories, Mumbai, India) were included. The diameter of the zones of inhibition was measured and recorded after 96 h of incubation at 25 °C, and the mean values were interpreted as sensitive (inhibition zone ≥ 10 mm) [34].

Minimum inhibitory concentration (MIC) values were determined using different concentrations (0.1, 0.32, 1, 3.2, and 5 mg/mL) of both AzA-TEs and free AzA as previously described [35,36]. The MICs of itraconazole (Janssen Research Foundation Beerse, Belgium) were determined according to Clinical Laboratory Standards Institute M38 guidelines [37]. The MIC_50_ and MIC_90_ values, which, respectively, prevented the growth of 50% and 90% of the tested isolates [38].

##### In Vivo Evaluation of Antidermatophyte Activity of AzA-TEs

The effectiveness of the optimized AzA-TEs formula in comparison with the free AzA and itraconazole for the treatment of dermatophytosis was investigated in two infected guinea pig models. For establishing infection, in the two models, with *M. canis* and *T. mentagrophytes*, hair from the mid-dorsal region (diameter of 1.50 cm) of 60 guinea pigs (weight 250–300 g) was shaved using a manual razor. Then, 50 µL of *M. canis* or *T. mentagrophytes* suspension (10^7^ cells/mL) was applied to the shaved skin. On the third day following inoculation, antifungal therapy was started on shaved regions and continued for 12 days [39].

In each model, guinea pigs were divided into five groups (6 animals/group): group 1 (G1); infected untreated control group, G2; infected and treated with 10 mg/kg itraconazole once daily by oral gavage [39]. AzA-TEs gel (5 mg/kg) (G3), AzA gel (G4), and plain gel (G5) were applied topically twice a day.

The infected and treated animals were evaluated clinically on days 3, 7, 10, and 14 post-treatment (PT) by scoring lesions (redness, scales, and hair loss) and mycologically by microscopy and culture on days 3, 7, and 14 PT. Animals were kept under observation up to day 28 PT to evaluate the final clinical cure rate.

The inoculated areas were disinfected with 70% ethyl alcohol and skin scrapings and hair samples were collected using a sterile toothbrush for subsequent microscopical examination using 20% potassium hydroxide (KOH) and culture on mycobiotic agar medium slants and dermatophyte test medium (DTM) plus dermato supplement (Himedia, India). Cultures were incubated at 25 °C for four weeks and observed twice a week. Based on macro- and micro-morphological features, isolates were identified to the species level. If cultures of the collected samples from the infected regions were negative, the animals were considered mycologically cured [40].

#### 2.2.7. Data Analysis

Data were edited in Microsoft Excel (Microsoft Corporation, Redmond, WA, USA). Exact Wilcoxon test was used to compare untreated versus treated animals per day (3, 7, 10, and 14 PT) regarding the clinical score. The effect of the treatments was evaluated for each day (3, 7, and 14) by the frequency of positive microscopy and cultures in treated animals versus untreated control group according to the fisher exact test. Figures were fitted by the Graph-Pad Prism software 5.0 (Graph Pad, San Diego, CA, USA). Statistical significance was set at *p*-value less than 0.05.

## 3. Results and Discussion

### 3.1. Effect of Formulation Variables on the Observed Responses

#### 3.1.1. Particle Size and Size Distribution

As seen from Table 3, the mean particle size ranged from 219.8 to 403.5 nm for formulae F3 and F4, respectively. Formulations F4 and F1 showed the widest and narrowest particle size distributions with PDI values of 0.249 and 0.414, respectively. Only one formula exceeded the PDI value of 0.4 and none exceeded the value of 0.5. The obtained values are acceptable and indicate relatively homogenous vesicular size distribution). According to Soleimanian et al., a PDI higher than 0.5 is not considered acceptable and reflects heterogeneous, wide size distribution values [41]. Regarding the particle size, a smaller size was achieved using a higher ethanol concentration. The model that best fits the particle size data is a quadratic model and the equations that best describe the model for different surfactants are:

PS = SAA type Labrafil + 364.38750 + 4.53750 SAA:Lecithin − 3.96625 Ethanol Conc

PS = SAA type SDC + 396.96250 + 4.53750 SAA:Lecithin + 3.96625 Ethanol Conc

**Figure 1:**
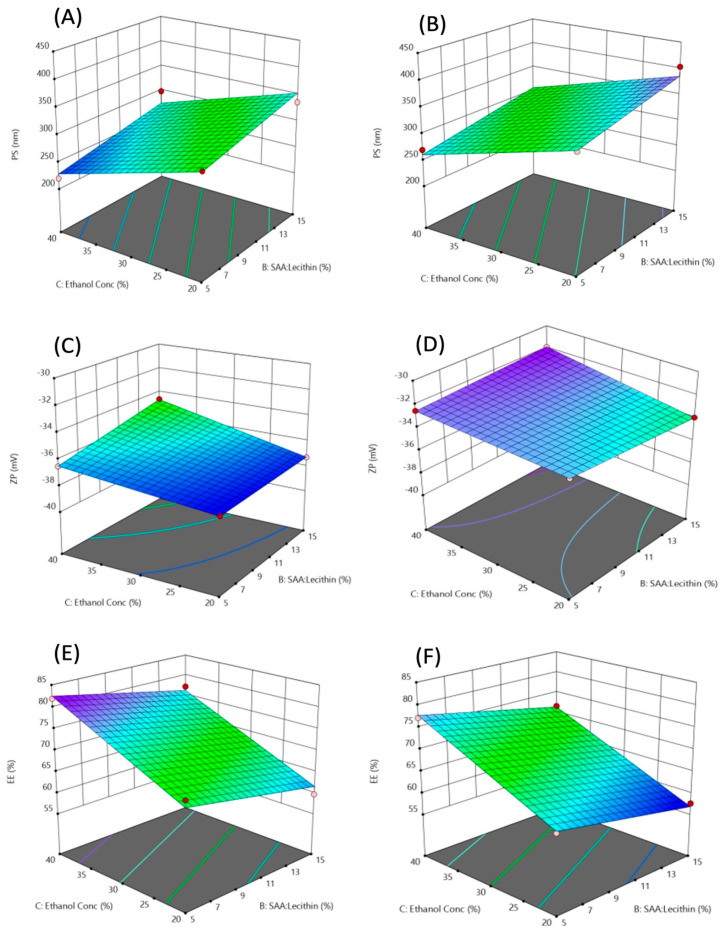
3D-response surface plots showing the effect of the independent variables on PS (**A**,**B**), ZP (**C**,**D**), and EE% (**E**,**F**). (**A**,**C**,**E**) where the used SAA is Labrafil, (**B**,**D**,**F**) where the used SAA is SDC.

The model was found significant (*p*-value 0.0170) (Figure 1A,B). Regarding the variables studied, it was found that increasing the ethanol concentration led to a decrease in the particle size. This effect might be due to the interaction between the ethanol and lipid bilayer [42]. Another explanation was presented by Vasanth and colleagues who suggested that high ethanol concentration causes interpenetration of the Lecithin hydrocarbon chain, which leads to a further reduction in the thickness of the membrane of the TEs vesicles and causes a decrease in mean particle size [43]. This was in agreement with Salem et al., who found a generalized reduction in the mean vesicle size by increasing ethanol content from 10 to 30%. Ahmed et al. found the same antagonistic effect of ethanol concentration; they offered an additional explanation that upon increasing the ethanol content, it causes a reduction in the main transition temperature of the phospholipids, which leads to partial fluidization of the TEs vesicles and the formation of small nanovesicles [44]. The same observation was found during the preparation of ethosomes by Limsuwan et al., who found that the highest ethanol concentration led to a smaller size of the prepared ethosomes [45]. High concentrations of ethanol—higher than 40%—were not attempted as it was mentioned previously that higher concentrations as 45% lead to the failure of vesicle formation or rapture of formed vesicles [46]. The other factor that was significant on the PS of the vesicles was the SAA to Lecithin ratio. It was found that decreasing the ratio led to a decrease in the PS. The same finding was observed by Qushawy et al., who found that the SAA:Lecithin ratio of 10:90 resulted in a smaller size of transfersomes compared to the 20:80 ratio [47]. Vasanth et al., who studied the same variable on transfersomes stated that the higher concentration of soy lecithin led to smaller vesicle size [43]. It is worth mentioning that some other studies found that this effect was insignificant on the vesicular size of TEs [27] and transfersomes [48]. No significant effect was found when either SDC or Labrafil M 1944 Cs were used on the vesicular size.

#### 3.1.2. Zeta Potential

As expected from the composition, the TEs have negative charges due to the presence of oleic acid and the phosphate group in soy lecithin. As seen from Table 3, all prepared formulations show a ZP value over −30, which indicates high stability and a low tendency to agglomerate using all tried ingredients in all levels of the variables used. This leads to higher stability of TEs dispersion.

ZP = SAA type Labrafil − 37.9 − 0.135 SAA:Lecithin + 0.0125 Ethanol Conc + 0.008 SAA:Lecithin × Ethanol Conc

ZP = SAA type SDC − 32.15 − 0.255 SAA:Lecithin − 0.0175 Ethanol Conc + 0.008 SAA:Lecithin × Ethanol Conc

The model used was significant (*p*-value 0.027) (Figure 1C,D). It was mentioned by Ogiso et al. that the negative charge of the zeta potential of ethosomal systems is attributed mostly to the high ethanolic content in these nanovesicles. Ethanol provides negative charges to the polar head groups of the phospholipids that would create an electrostatic repulsion [49]. Formulations containing Labrafil M 1944 CS with the chemical formula mono-, di- and triglycerides, and PEG-6 (MW 300) mono- and diesters of oleic (C18:1) acid showed higher ZP than those containing SDC as surfactant. No significant effect of the SAA:Lecithin ratio on the ZP.

#### 3.1.3. Entrapment Efficiency

High entrapment efficiencies of AzA up to 81.9% for formulation F3 were obtained. The least EE% value was in F4 with only 57.6% AzA entrapped. The model to optimize the AzA-TEs was highly significant (*p*-value = 0.0004) (Figure 1E,F). In addition, all variables showed a significant effect on the EE% of the prepared TEs. This could be explained by the co-solvent effect of the ethanol which has a high effect in increasing the solubility of lipophilic drugs—such as AzA—in the polar phase of the TEs. This also allows an additional amount of AzA in the aqueous core of the nano transethosomal dispersion to be accommodated [50]. Another explanation that was presented by Salem et al. is that the solubilization effect of ethanol increases the fluidity of the membrane of the TEs vesicles leading to a more entrapped AzA [51]. An interesting finding was obtained as decreasing SAA:Lecithin ratio resulted in higher EE%. In a lower ratio, higher lecithin concentration in the vesicular membrane might increase the entrapment of the hydrophobic AzA. The obtained results were in good agreement with Balata et al., who found that increasing surfactant concentration was accompanied by a decrease in EE%. They suggested that this might be due to the increased efficiency of surfactant incorporation within the lipids forming a more permeable vesicles membrane, hence lowering the EE% [48,52]. Formation of mixed micelles upon the addition of higher SAA concentrations is another explanation for lower EE% as they are more rigid and smaller in size [53]. Labrafil M 1944 SC with an HLB of 9 was found to achieve higher EE% compared to SDC. SDC is an anionic surfactant with a high HLB of 16. An explanation of the lower EE% in formulations containing SDC can be offered by discussing the findings of Amnuaikit et al., who found that high EE% of transfersomes contains the cationic drug phenylethyl resorcinol using SDC as SAA. They concluded that the high EE% was due to electrostatic attraction between the negatively charged drug and the positively charged SDC [54]. Using the same concept, the anionic AzA showed electrostatic repulsion with the anionic SDC resulting in the expulsion of the drug from the transethosomal vesicles, which lead to decreased EE%. The model equations for each surfactant type were

EE = SAA type Labrafil + 56.575 − 0.635 SAA:Lecithin + 0.7275 Ethanol Conc

EE = SAA type SDC + 51.825 − 0.635 SAA:Lecithin + 0.7275 Ethanol Conc

### 3.2. Optimization of Formulation Variables

Based on the analysis of the studied variables, the optimization was carried out using the desirability function approach. When several responses were evaluated in an experimental design, the optimum responses reached individually for each factor did not coincide in a single run in all cases. To solve multiple response problems, different statistical methods can be used; one of the most commonly used methods is the desirability function [55]. To find the most desirable formulation fulfilling all constraints of all studied variables, a weight factor of 1 was chosen for all individual desirabilities in this work. The best selected solution with the highest desirability (0.920) was found to be the same as the composition of F3. The selected solution and the predicted and practically performed formulations are listed in Table 4. Labrafil M 1944 SC was used as SAA with a ratio to lecithin of 5:95, respectively, and 40% ethanol concentration. As seen from Table 4 a small acceptable residual error was obtained indicating the validity of the model for the preparation of AzA-TEs. The selected formula was further characterized and investigated both in vitro, ex vivo, and in vivo for antidermatophyte effect.

#### 3.2.1. Transmission Electron Microscopy

**Figure 2:**
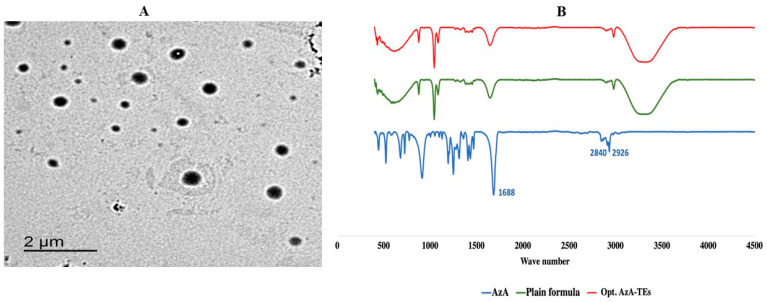
Characterization of the optimized formula; (**A**) Transmission electron microscopy of the optimized AzA-TEs; (**B**) FTIR spectra of the free AzA, plain formula, and optimized AzA-TEs.

As seen in Figure 2A, the AzA-TEs vesicles are spherical in shape and homogenous in size. They are present as separate entities without agglomeration.

#### 3.2.2. Fourier Transform Infrared (FTIR) Spectroscopy

Figure 2B showed the FTIR spectrum of the free AzA, plain formula, and the optimized AzA-TEs. The FTIR spectrum of the AzA reveals typical bands coming from the aliphatic chain at 2926 cm^−1^ and 2840 cm^−1^. There is also a band of high intensity coming from two free carboxyl groups at about 1700 cm^−1^ situated at the end of the molecule [56]. FTIR spectrum of AzA-TEs showed that the characteristic absorption peaks of AzA disappeared indicating the drug encapsulation within the nanovesicles. In addition, the peaks of the AzA-TEs optimized formula and the plain drug free formula were identical, which confirms that AzA was successfully encapsulated in the nanovesicles [57].

#### 3.2.3. In Vitro Release of AzA from the Optimized Formula

**Figure 3:**
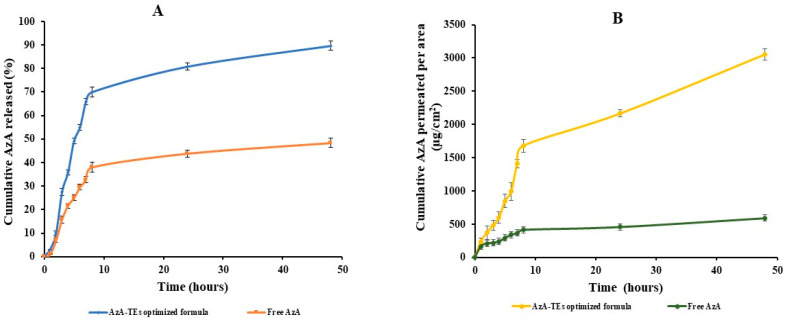
In vitro and ex vivo characterizations of the optimized formula; (**A**) In vitro drug release of the free AzA and the optimized AzA-TEs; (**B**) Ex vivo fluxes of the free AzA and the optimized AzA-TEs.

The in vitro release study of AzA-TEs optimized formula and free AzA was performed in a release media of phosphate-buffered saline pH 7.4 and ethanol (30%) and the data are plotted in Figure 3A. The attained data demonstrated that the optimized formulation reached 89% cumulative release after 48 h, in comparison to 45% only of the free AzA. The release behavior of AzA from AzA-TEs optimized formula showed a controlled release manner during the 48 h of the study in comparison to the free AzA that exhibited an uncontrolled release behavior. The dissolution rate of AzA-TEs was found to be faster than those of free AzA. The increase in the amount released could be due to the small PS of the TEs nanovesicles that led to the higher surface area, and hence improved dissolution rate. Additionally, the free drug solubility could be improved by reducing the PS according to the Oswald–Freundlich equation [58]. The abovementioned findings are in accordance with a study that used telmisartan ethosomal gel [27].

#### 3.2.4. Ex Vivo Permeation of AzA from the Optimized Formula

The cumulative amount of AzA permeated from AzA-TEs optimized formula compared to the free AzA after 48 h is depicted in Figure 3B. It was detected that the amount of AzA permeated from the optimized formula (3056 µg/cm^2^) was higher than the amount permeated from free AzA suspension (590 µg/cm^2^) after 48 h, which may be due to that AzA can overcome the SC barrier and reach the deeper layers of the skin more efficiently than AzA in its free form. This behavior may be attributed to the presence of ethanol in the TEs nanovesicles which act as penetration enhancer and thus imparts flexibility to TEs and therefore is responsible for the high penetration [59]. It was reported that TEs employ pull and push effects on the intercellular interface of the SC cells and thus could enhance the drug penetration [59]. The push effect is a thermodynamic effect of ethanol evaporation, and the pull effect is a result of ethanol fluidizing SC lipids, which results in the formation of additional penetration pathways [30,59]. It is also worth noting that AzA-TEs obtained higher permeability parameters with a Jmax of 35.95 μg/cm^2^/h compared to 6.94 μg/cm^2^/h of free AzA with an ER of 5.18.

### 3.3. Evaluation of Antidermatophyte Activity

#### 3.3.1. In Vitro Evaluation of Antidermatophyte Activity of AzA-TEs

This study records the antidermatophyte activity of an optimized AzA-TEs formula against three dermatophyte species. It exhibited the maximum inhibition of all strains tested, while the free AzA showed the minimum growth suppression. It was found that AzA-TEs liquid dispersion induces a significantly greater inhibition of the tested dermatophyte species than free AzA, whereas the mean inhibition zone diameters were 27.9 ± 0.15, 25.17 ± 0.05, and 26.15 ± 0.35 mm versus 16.8 ± 0.7, 15.83 ± 0.73, and 19 ± 0.21 for *T*. *rubrum*, *T. mentagrophytes*, and *M*. *canis*, respectively.

The MIC values obtained with the AzA-TEs formula were lower than those of free AzA for all test dermatophyte species (MIC_90_ was 0.01% vs. 0.32% for *T. rubrum* and 0.032% for *T*. *mentagrophytes* and *M. canis* vs. 0.56%). MIC range for itraconazole was 0.25–2, 0.03–1, and 0.125–1 µg/mL for *T. rubrum*, *T. mentagrophytes*, and *M. canis*, respectively.

This supports the findings of an earlier study that reported the antifungal activity of AzA (0.56%) against dermatophytes and *Scopulariopsis brevicaulis*. Moreover, 0.032% concentration of AzA did inhibit the strains tested, and testing the in vivo antifungal activity of this agent was encouraged [35]. Since *T. mentagrophytes* and *M. canis* produce acute inflammatory infections which are easy to clinically evaluate [60,61], we have established two dermatophytosis infection models using both species to be evaluated in vivo.

AzA-TEs showed an effective antifungal effect, which may be due to their unique subcellular size which can effectively increase the distribution of antifungal agents in fungal cells; therefore, greater antifungal activity was achieved [62].

#### 3.3.2. In Vivo Evaluation of Antidermatophyte Activity of AzA-TEs

**Figure 4:**
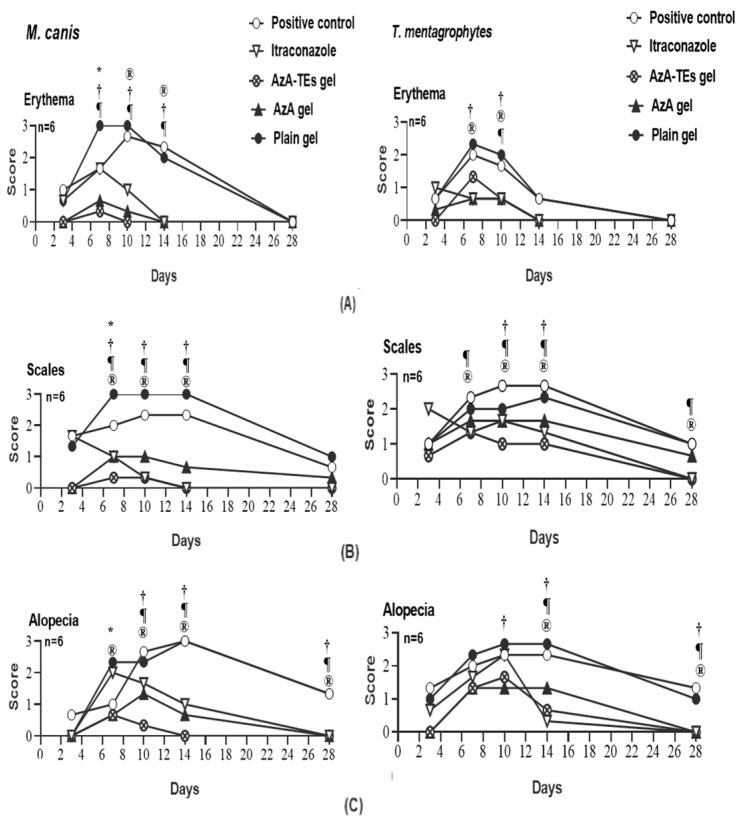
Means of erythema (**A**), scales (**B**), and alopecia (**C**) scores in *Microsporum canis* and *Trichophyton mentagrophytes* infected guinea pigs in different groups. * *p* < 0.05 (plain gel); † *p* < 0.05 (AZA gel); ^®^ *p* < 0.05 (itraconazole); ¶ *p* < 0.05 (AzA-TEs gel) versus untreated positive controls. *p*-value was estimated using exact Wilcoxon test.

As presented in Figure 4, the infection progressed in the untreated control group (G1) and plain-gel-treated group (G5) with increasing lesion scores, while decreasing scores of erythema, scales, and alopecia were observed in the treated groups (G2, G3, and G4). Maximum response of erythema in G1 and G5 was on day 10 in the *M. canis* model and day 7 in the *T. mentagrophytes* model. While maximum responses of scales and alopecia were on days 10–14 in G1 and G5 in the *T. mentagrophytes* model and on day 14 maximum alopecia was observed in the *M. canis* model. There is a significant (*p* < 0.05) lower erythema and lesion scores in the AzA-TEs gel-, AzA gel-, itraconazole-, and plain-gel-treated groups versus the untreated control group (Figure 4).

Animals in the treated groups were positive in direct microscopy during the 14 days observation period, whereas most of the culture from AzA-TEs gel (G3) and AzA gel (G4)-treated groups in the *M. canis* model were negative during the 14-day observation period (Table 5). Although five cultures from treated groups in the *T. mentagrophytes* model were negative on day 3 and day 7 PT, two cultures from itraconazole and AzA gel treated animals were positive on day 14 PT (mycological cure rate was 66.76%). Nonetheless, the mycological cure rate was 83% in AzA-TEs gel-treated group after 14 days from the onset of treatment. Cultures of untreated control and plain-gel-treated animals were persistently positive in both *T. mentagrophytes* and *M. canis* models. Therefore, there is a significant difference between treated groups versus untreated control and plain-gel-treated ones.

Guinea pigs are used in animal models for dermatophytosis because they are predisposed to cutaneous fungal infections with clinical characteristics similar to those found in humans [63,64]. In *M. canis-* and *T. mentagrophytes*-infected animals, significantly lower clinical scores were observed in treated as compared to untreated animals (Figure 4). The ability of AzA-TEs gel (G3) and AzA (G4) to reduce the erythema, scales, and hair loss in the treated animals could be directly related to its anti-inflammatory and antikeratinizing activity (decrease in the size and quantity of keratohyalin granules and tonofilament bundles due to alteration of epidermal keratinization, which is especially detrimental to the terminal phases of epidermal differentiation) [65].

Most treated animals were culture negative, but some animals remained microscopy positive on day 14 PT (Table 5). This could be due to the non-viable conidia and hyphae that are detected using microscopy and would not grow on culture [35,66]. The mycological cure rate in *M. canis* infected and treated animals after 14 days from onset of treatment was 83% but, in the *T. mentagrophytes* model, the cure rate was 83% in AzA-TEs gel-treated group and 66.76% in itraconazole and AzA groups. Similarly, cure rates after treatment with antifungal agents ranged from 80–100% were reported in other studies [40,67]. However, the failure of mycological cure was observed in the *T. mentagrophytes* infected animals [35,68].

**Figure 5:**
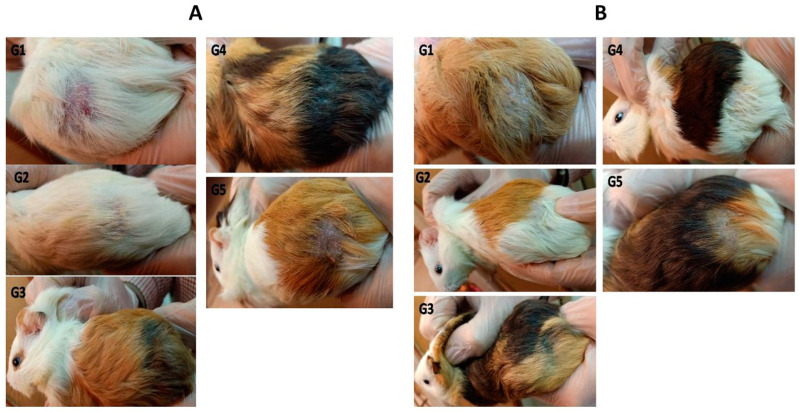
*M. canis* (**A**) and *T. mentagrophytes* (**B**) infected guinea pig models at day 14 post-treatment. G1: infected untreated control group; G2: infected and treated with 10 mg/kg itraconazole once daily through oral gavage; G3: AzA-TEs gel-treated group (5 mg/kg); G4: AzA gel, and G5: plain-gel-treated groups topically twice a day.

The superiority in the anti-fungal activity of the AzA-TEs gel formulation over other formulations (Figure 5) may be attributed to the high flexibility of the TEs, helping its penetration through the fungal cell wall and preventing ergosterol synthesis led to fungal cell membrane lysis and cell death [69]. Moreover, the lipid nanoparticles have small size that could enhance the presence of the nanoparticles to be in direct contact with the SC and confirms the entry of encapsulated drugs into the skin [70]. In addition, the extra potential of ethanol could kill organisms by denaturing their proteins and dissolving their lipids, apart from skin fluidization and penetration [71].

## 4. Conclusions

The TEs formulations proved efficacious in different skin conditions, skin cancer and cosmetics. They ensure better skin penetration and hence improved both local skin retention and transdermal action. Despite these advantages, the optimization of TEs composition is quite challenging. Several factors affect the formulation such as the nature of the drug and formulation variables. In the current study, AzA-TEs were successfully prepared using the thin film hydration method. The formulation variables for AzA-TEs were optimized and the optimized formulation showed a small size, high ZP and EE%, a prolonged release, and successful skin penetration. The obtained results from in vitro testing and in vivo models essentially imply that the therapeutic effects of AzA-TEs in dermatophytosis are directly mediated by their antidermatophyte activity. The TEs are promising carriers for enhancing the antidermatophyte activity of AzA by deep penetration through skin layers. In future, further studies related to human activity, stability, and toxicity should be performed. In addition, scaling up to an industrial scale should be studied and optimized. These results provide an important perspective on developing a safe and efficient antifungal drug for treating dermatophytoses.

## Figures and Tables

**Table 1:** Full factorial design (2^3^) used for preparation and optimization of AzA-TEs.

Independent Variables	Low	High
X1: SAA type	Labrafil	SDC
X2: SAA:Lecithin ratio X3: Ethanol concentration%	15:85 20	5:95 40
Dependent Variables	Desirability Constraints	
Y1: PS Y2: ZP	Minimize Maximize	
Y3: EE%	Maximize	

Abbreviations: AzA: Azelaic acid, TEs: Transethosomes; SAA: Surfactant; SDC: Sodium deoxycholate; PS, particle size; ZP, zeta potential, and EE%, entrapment efficiency percentage.

**Table 2:** Composition of the prepared TEs loaded with AzA.

Formulae Code	AzA Amount (mg)	Oleic Acid Amount (mg)	SAA Type	SAA: Lecithin Ratio	Ethanol Concentration (%)
F1	50	10	Labrafil	5:95	20
F2	50	10	SDC	15:85	40
F3	50	10	Labrafil	5:95	40
F4	50	10	SDC	15:85	20
F5	50	10	Labrafil	15:85	20
F6	50	10	SDC	5:95	40
F7	50	10	SDC	5:95	20
F8	50	10	Labrafil	15:85	40

Abbreviations: AzA: Azelaic acid; TEs: Transethosomes; SAA: Surfactant; SDC: Sodium deoxycholate.

**Table 3:** Observed responses for the prepared TEs formulations.

	PS (nm)	PDI	ZP (mV)	EE (%)
F1	309.0 ± 7.8	0.414 ± 0.067	−37.5 ± 0.35	69.5 ± 2.0
F2	281.2 ± 3.7	0.390 ± 0.002	−31.9 ± 0.23	71.6 ± 1.3
F3	219.8 ± 4.7	0.372 ± 0.015	−36.5 ± 0.73	81.9 ± 1.4
F4	403.5 ± 8.5	0.249 ± 0.022	−33.9 ± 0.77	57.6 ± 0.8
F5	335.9 ± 3.9	0.292 ± 0.029	−37.3 ± 0.61	59.7 ± 0.9
F6	270.2 ± 8.0	0.375 ± 0.013	−34.5 ± 1.03	77.2 ± 3.1
F7	338.5 ± 1.0	0.346 ± 0.014	−33.0 ± 0.66	62.8 ± 1.1
F8	298.4 ± 1.6	0.269 ± 0.013	−34.6 ± 0.78	77.1 ± 2.4

Abbreviations: TEs: Transethosomes; PS, particle size; ZP, zeta potential; and EE%, entrapment efficiency percentage.

**Table 4:** Validation of the optimization model using the desirability function.

	Independent Variables			Responses			Desirability
	SAA Type	SAA Ratio	Ethanol Conc.	PS	ZP	EE%	
Predicted formula	Labrafil	5.0	40.0	228.425	−36.475	82.5	0.920
Practically prepared formula	Labrafil	5.0	40.0	219.8	−36.5	81.9	
Residual error (%)				3.78%	0.07%	−0.73%	

Abbreviations: SAA, Surfactant; PS, particle size; ZP, zeta potential; and EE%, entrapment efficiency percentage.

**Table 5:** Mycological evaluation of dermatophytosis treatments in a guinea pig model.

		Day 3					Day 7					Day 14				
		G1	G2	G3	G4	G5	G1	G2	G3	G4	G5	G1	G2	G3	G4	G5
*T. mentagrophytes*	M	6	6	6	6	5	5	4	1 †	4	5	6	2 †	1 †	2 †	6
	C	5	1 †	1 †	1 †	4	6	1 †	1 †	1 †	6	6	2 †	1 †	2 †	6
	M&C	5	1 †	1 †	1 †	4	6	1 †	1 †	1 †	6	6	2 †	1 †	2 †	6
*M. canis*	M	6	6	6	6	6	6	6	3	3	6	6	1 †	3	3	6
	C	3	2	0	0	3	6	4	1 †	1 †	6	6	1 †	1 †	1 †	6
	M&C	3	2	0	0	3	6	4	1 †	1 †	6	6	1 †	1 †	1 †	6

Treated groups versus positive controls were compared using Fisher’s exact test per day. † differs significantly with positive control *p* < 0.05. M: microscopy; C: culture; G1: infected untreated control group; G2: infected and treated with 10 mg/kg itraconazole once daily by oral gavage; G3: AzA-TEs gel- treated group (5 mg/kg); G4: AzA gel, and G5: plain-gel-treated groups topically twice a day.

## Data Availability

The datasets generated or analyzed during the current study are available from the corresponding author upon reasonable request.
